# Plant Biodiversity Drivers in Brazilian *Campos Rupestres*: Insights from Phylogenetic Structure

**DOI:** 10.3389/fpls.2017.02141

**Published:** 2017-12-19

**Authors:** Daniela C. Zappi, Marcelo F. Moro, Thomas R. Meagher, Eimear Nic Lughadha

**Affiliations:** ^1^Biodiversity and Ecosystem Services Team, Instituto Tecnológico Vale, Belém, Brazil; ^2^Coordenação de Botânica, Museu Paraense Emílio Goeldi, Belém, Brazil; ^3^Instituto de Ciências do Mar (Labomar), Universidade Federal do Ceará, Fortaleza, Brazil; ^4^School of Biology, University of St Andrews, St Andrews, United Kingdom; ^5^Conservation Science Department, Royal Botanic Gardens, Kew, Richmond, United Kingdom

**Keywords:** *campo rupestre*, *canga*, eastern Brazil, phylogenetic clustering, rupestrian grasslands, substrate, vegetation types

## Abstract

Old, climate-buffered infertile landscapes (Ocbils) have attracted increasing levels of interest in recent years because of their exceptionally diverse plant communities. Brazil’s *campos rupestres* (rupestrian grasslands) are home to almost 15% of Brazil’s native flora in less than 0.8% of Brazil’s territory: an ideal study system for exploring variation in floristic diversity and phylogenetic structure in sites differing in geology and phytophysiognomy. We found significant differences in floristic diversity and phylogenetic structure across a range of study sites encompassing open vegetation and forest on quartzite (FQ) and on ironstone substrates, commonly termed *canga*. Substrate and physiognomy were key in structuring floristic diversity in the Espinhaço and physiognomy was more important than substrate in structuring phylogenetic diversity, with neither substrate nor its interaction with physiognomy accounting for significant variation in phylogenetic structure. Phylogenetic clustering was significant in open vegetation on both *canga* and quartzite, reflecting the potential role of environmental filtering in these exposed montane communities adapted to multiple environmental stressors. In forest communities, phylogenetic clustering was significant only at relatively deep nodes of the phylogeny in FQ while no significant phylogenetic clustering was detected across forest on *canga* (FC), which may be attributable to proximity to the megadiverse Atlantic forest biome and/or comparatively benign environmental conditions in FC with relatively deep, nutrient-rich soils and access to edaphic water reliable in comparison to those for open vegetation on *canga* and open or forest communities on quartzite. Clades representing relatively old lineages are significantly over-represented in *campos rupestres* on quartzite, consistent with the Gondwanan Heritage Hypothesis of Ocbil theory. In contrast, forested sites on *canga* are recognized as Yodfels. To be effective, conservation measures must take account of the distinct communities which are encompassed within the broad term *campos rupestres*, and the differing vulnerabilities of Ocbils and Yodfels.

## Introduction

Found in different continents worldwide, old (ancient), climate-buffered, infertile landscapes (termed Ocbils) harbor high endemism and diversity, as exemplified by the Australian *kwongkan*, South African *fynbos*, Guayana shield *tepuis* (Hopper, 2009). These habitats are believed to have persisted over very long timeframes on the scale of many millions of years due to the geological stability and climatic buffering of their locations. The specialized environmental conditions of such habitats, and the long timeframe over which they have persisted, represent a useful system in which to explore the collective ecological and evolutionary response of plants that exist in them.

**FIGURE 1:**
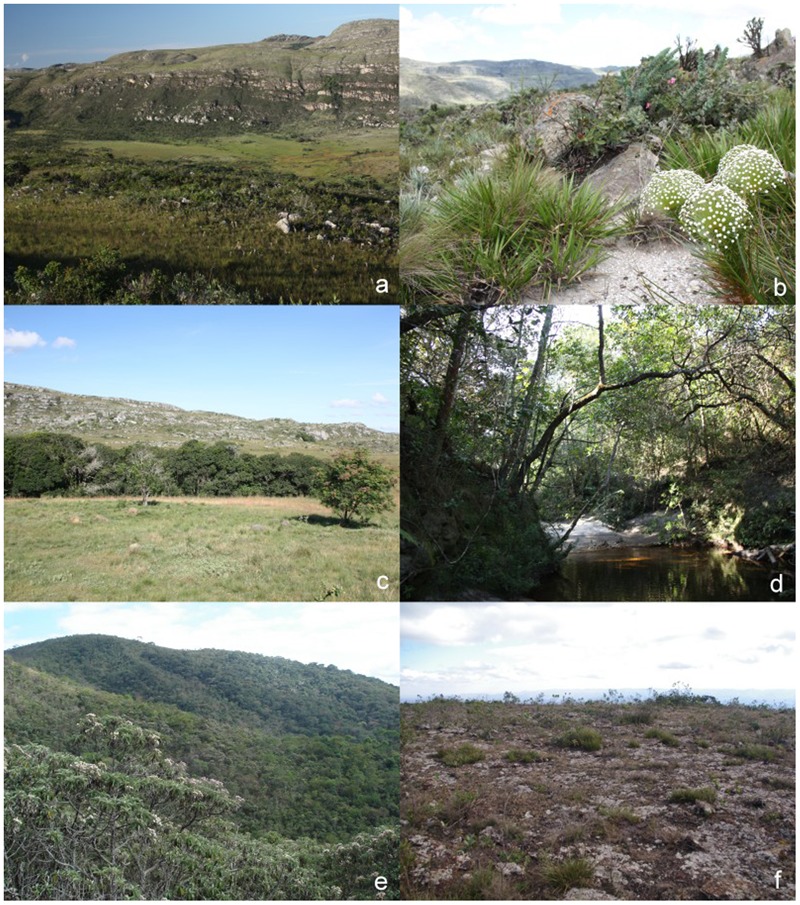
*Campos rupestres* with different substrates in Minas Gerais. **(a)** Landscape of the Serra do Cipó, Mun. Santana de Pirapama, showing different open quartzite (OQ) vegetation types; **(b)** OQ - close-up of ‘cerrado-rupestre’ with Eriocaulaceae in the foreground at the Serra do Cipó; **(c)** forest on quartzite (FQ) - view of a forest grove in a matrix of open vegetation, Serra do Cipó; **(d)** FQ - Inside a riverine forest, Serra do Cipó; **(e)** FC - forest formation on *canga* at the Serra da Gandarela, Mun. Rio Acima; **(f)** open vegetation on *canga* (OC) - view of *canga* field at the Serra de Capanema, Mun. Catas Altas (photos **a–d** William Milliken; **e–f** Pedro L. Viana).

Brazilian rupestrian habitats, locally known as ‘*campos rupestres*,’ are an example of a long-standing climate-buffered infertile landscape that contains many endemic species and lineages, which are adapted to very shallow and nutrient-poor soils (Silveira et al., 2016). The highlands in which most *campos rupestres* occur are situated primarily in the states of Minas Gerais and Bahia, with outliers in Goiás and Tocantins. Thus, they are set within three major Brazilian biomes: to the Southeast, they are embedded in the westernmost part of the Atlantic Rainforest in the state of Minas Gerais, while to the north and west of Minas Gerais state they are found within the Cerrado savannas. In Bahia state, the *campos rupestres* occur within the Caatinga semi-arid biome. The *campos rupestres* of the highlands of the Espinhaço range, which extends through Minas Gerais and Bahia, are the better botanically explored and have long been recognized as sites of exceptional biodiversity and endemism (Stannard, 1995; Giulietti et al., 1997) and, more recently, as fulfilling several of the criteria used to characterize Ocbils (Hopper et al., 2016; Silveira et al., 2016). While *campos rupestres* are particularly associated with the Espinhaço range, they also occur on even more ancient rock formations in the Quadrilátero Ferrífero, to the south of the Espinhaço (Schaefer et al., 2016). Along the Espinhaço range, different vegetation types can be found (**Figure 1**). Forests and savannas mostly grow at lower altitudes or along the rivers (**Figures 1c,d**), while patches of *campo rupestre* grasslands develop at the more exposed sites of mountain tops (**Figure 1a**). Occurring on either quartzitic, arenitic or ironstone substrates, typically at altitudes >900 m above sea level, *campos rupestres* are estimated to occupy 66,450 km^2^ and to be home to over five thousand known species (Silveira et al., 2016), of which over two thousand are recorded as endemic to this habitat type and occur over a relatively small area (Brazil Flora Group [BFG], 2015).

The high levels of floristic diversity and endemism for which *campo rupestre* vegetation is renowned have often been ascribed to the disjunct (mosaic) distribution of *campo rupestre* sites and resulting floristic influences from other habitats, especially savannas (Giulietti et al., 1997; Neves et al., 2018). Recent recognition of *campos rupestres* as an Ocbil has provided a wider theoretical framework within which the ecology and evolution of their extraordinary plant diversity is being interpreted in a global context and over extended geological timescales (Silveira et al., 2016). Two recent in-depth reviews (Hopper et al., 2016; Silveira et al., 2016) have shown several of the predictions of Ocbil theory to be clearly applicable to *campos rupestres*, and further studies of specific subsets of the *campo rupestre* flora are adding to this emerging picture. For example, in a study of 210 plant species from *campo rupestre* Dayrell et al. (2017) found that the majority (62.5%) had non-dormant seeds, the highest proportion of non-dormancy reported for any vegetation type to date and consistent with predictions of reduced dispersability in Ocbils. Although this study encompassed representatives of 20% of the plant families represented in *campo rupestre*, fewer than 5% of the plant species known from *campo rupestre* were treated, illustrating the scale of the challenge in understanding this exceptional flora.

While *campo rupestre* can be associated with different substrates, such as quartzite, ironstone or sandstone, the diversity and specificity of iron-ore plant communities and their occurrence on iron-rich substrates of enormous mining interest means that they are amongst the most threatened vegetation types in Brazil (Jacobi et al., 2007). Unprecedented pressure on natural resources such as iron-ore make Brazil second only to Australia in the quantity of seaborne iron-ore it exports to China (Lawrence and Nehring, 2015). There are three major iron-mining districts in Brazil, namely Carajás in Pará, Urucum in Mato Grosso, and the *campos rupestres* associated with the Quadrilátero Ferrífero (Iron Quadrangle) in Minas Gerais, where the iron-rich substrate is known as *canga*. Comparative studies of the flora of *campos rupestres* on quartzite and *canga* have shown strong correlations between plant species abundance, vegetation cover, coverage of individual species and soil properties between these two habitats (Vincent and Meguro, 2008; Messias et al., 2012a, 2013). Tolerance to (and accumulation of) high metal concentration has evolved independently in different phylogenetic lineages (Broadley et al., 2001), allowing plants of different lineages coming from various ecosystem types to occupy and diversify in iron-rich *canga* habitats. Such tolerance may represent an environmental filter shaping *canga* communities in addition to the many factors they share with other *campo rupestre* environments such as intense solar radiation, high evapotranspiration, large daily temperature variations and soils characterized by poor water retention and low nutrient availability (Jacobi et al., 2007).

The availability of increasingly comprehensive plant phylogenies has enabled exploration of the roles of environmental filtering (selection imposed by environmental extremes that favor specific traits), and niche conservatism, in which such traits tend to be shared among species that are closely related phylogenetically (Webb et al., 2002; Cavender-Bares et al., 2009). Strong environmental drivers, such as specialized substrates, are a major factor in shaping local biodiversity (e.g., Webb, 2000). Thus, we would expect *campos rupestres* on quartzite and *canga* to differ in terms of the impacts of environmental filtering on the floristic composition and phylogenetic structure of their communities.

To date the majority of studies examining phylogenetic structure over environmental gradients in tropical plant communities have focused on climate, with only a few examining other factors such as soil fertility and even these tend to be focused on tropical trees and palms (Lehtonen et al., 2015). Until recently, very few studies have focused on open vegetation formations, or on plant habits other than trees; but this is starting to change with studies on Caatinga (Moro et al., 2015), *campos gerais* (Moraes et al., 2016) and *campos rupestres* (Miazaki et al., 2015; Pugliesi and Rapini, 2015). Both of the latter studies focus on *campos rupestres* on quartzite; and, although they differ markedly in geographic scale and taxonomic scope, they both evidence phylogenetic clustering within quartzitic *campo rupestre* assemblages. Considering distribution records for a single family, the Apocynaceae, across the northern part of the Espinhaço range (widely known as the Chapada Diamantina), Pugliesi and Rapini (2015) found significant phylogenetic clustering which they attributed to niche conservatism and limited dispersal leading to *in situ* diversification and high density of microendemics. On a more local spatial scale, comparing the angiosperm communities on two sites within Itacolomi State Park in Minas Gerais state, Miazaki et al. (2015) concluded that environmental severity reduces phylogenetic diversity and increases phylogenetic clustering in *campo rupestre* vegetation, as predicted by the stress dominance hypothesis (Swenson and Enquist, 2007).

In this study we compare the composition of *campo rupestre* plant assemblages having different physiognomies (open and forest formations) and occurring on contrasting substrates (quartzite and *canga*) in the Espinhaço range and the Quadrilátero Ferrífero. We construct the first *campo rupestre* supertree (believed to be the first for any Ocbil) and use it to compare the phylogenetic structure of these contrasting assemblages. Based on earlier studies in *campos rupestres*, and consistent with Ocbil theory, we predicted that phylogenetic clustering would be prevalent in the assemblages analyzed. Assuming the prevalence of niche conservatism, consistent with Ocbil theory, we anticipated that the high levels of metals in *canga* might represent an additional environmental filter potentially resulting in accentuated phylogenetic clustering in *canga* assemblages. We also predicted significant over-representation of clades representing relatively old lineages, consistent with the Gondwanan Heritage Hypothesis of Ocbil theory (Hopper et al., 2016). We discuss our results in the context of recent publications demonstrating or inferring environmental filtering in *campos rupestres* (Negreiros et al., 2014; Miazaki et al., 2015; Pugliesi and Rapini, 2015) and those highlighting the *campo rupestre* ecosystem as a recently recognized Ocbil and a long-neglected conservation priority (Silveira et al., 2016; Neves et al., 2018).

## Materials and Methods

### Site Survey Collation

A literature search was conducted to locate published botanical studies that include species lists for locations in the Espinhaço range and the Quadrilátero Ferrífero. The bibliographic search was carried out in Scopus in October 2015, using the following search terms: ALL(*brazil* AND *floristic* OR *list* OR *checklist* AND “*campo rupestre”* OR *canga*) AND PUBYEAR > *1991* AND PUBYEAR < *2016.* This search yielded 296 papers. Analysis of their abstracts identified papers with comprehensive floristic lists (for angiosperms) for sites in Espinhaço range or the Quadrilátero Ferrífero as candidates for inclusion in our study. Details of all selected studies and all Angiosperm species reported therein were entered in a database developed in the ‘plotsamples’ module of Brahms software (BRAHMS, 2015). Plant nomenclature was checked against and manually updated to follow the Brazilian List of Plants and Fungi (Brazil Flora Group [BFG], 2015). Samples determined with *cf.* (e.g., *Croton* cf. *subferrugineus*, where cf. comes from the Latin verb *conferre*, meaning it is comparable to this species) in the original lists, and where no further reliable identification in the virtual herbaria consulted was found, were assigned to these taxa. Names that were qualified with ‘*aff.’* in the original lists (e.g., *Microlicia* aff. *decipiens*) were removed from this study because the use of *affinis* in this context indicates that the specimen resembles the species mentioned but is not referable to it.

A species list derived from the Brazilian List of Plants and Fungi (Brazil Flora Group [BFG], 2015) was imported into Brahms to enable automatic correction of synonyms collated from floristic papers to the currently accepted names in the Brazil Flora Group [BFG] (2015) database. During this step, the majority of records were matched or corrected automatically, while some were not recognized and a few were corrected manually. Infraspecific categories (subspecies, varieties and forms) were not taken into account but treated at species level.

Each floristic survey was then classified according to vegetation physiognomy (forest or open vegetation) and substrate (e.g., *canga*, quartzite) reported in the publication. Where a single study encompassed multiple vegetation types, information contained within the publication was used to subdivide the list into sampling sites which were more homogeneous with respect to vegetation type. We categorized the physiognomy of each site as “forest” for vegetation with a canopy or as “open vegetation” in the case of grassland or savanna. The Espinhaço is very complex in geology (Alkmim, 2012) and thus we also classified each site according to the main substrate on which plant communities were growing: quartzite, *canga*. When physiognomy or substrate was not reported by the authors of the study and we could not classify the site based on our own field experience, we tagged such information as “unknown.” The resulting database documented 10668 occurrences of 4234 species across 66 sampling sites (Supplementary Data 1.1).

For the analyses reported here, we excluded from our initial database all sites for which we could not determine the physiognomy of the vegetation or the substrate of the site, as well as all sites where anthropogenic disturbance was reported to be prominent. Reports of *campos rupestres* on limestone were also excluded because we had a small number of such studies to compare (only two) and specialists suggest *campo rupestre sensu stricto* does not occur on alkaline soils (Silveira, personal communication). From the initial database (Supplementary Data 1.1), we retained 47 sites (**Table 1** and Supplementary Data 1.2) and 2920 species, with a total of 6951 species records (Supplementary Data 1.2), of which 5 sites were classified as forest growing on *canga* (FC), 9 as forest on quartzite (FQ), 11 as open vegetation on *canga* (OC) and 22 as open vegetation on quartzite (OQ).

**Table 1:** Areas included in the present study and corresponding number of sites (shown in parentheses for vegetation and substrate).

Area	Reference	Number of sites	Vegetation	Substrate
Santana do Pirapama, MG (PIR)	Zappi et al., 2014	12	Open (9) Forest (3)	Quartzite (12)
Pico das Almas, BA (PAL)	Stannard, 1995	9	Open (6) Forest (3)	Quartzite (9)
Grão Mogol, MG (GMO)	Mello-Silva, 2009	9	Open (6) Forest (3)	Quartzite (9)
Pico do Itabirito, MG (ITA)	Teixeira and de Lemos Filho, 2013	2	Open (2)	*Canga* (1) Quartzite (1)
Serra do Condado, MG (SCO)	Pifano et al., 2010	3	Open (1) Forest (2)	*Canga* (3)
Barão de Cocais, MG (COC)	Mourão and Stehmann, 2007	1	Open (1)	*Canga* (1)
Serra da Calçada – Brumadinho, MG (CAL)	Viana and Lombardi, 2007	3	Open (2) Forest (1)	*Canga* (3)
Serra de Antônio Pereira – Ouro Preto, MG (SAP)	Messias et al., 2012b; Scalon et al., 2012	4	Open (4)	*Canga* (4)
Serra do Rola Moça, MG (RO1, RO2)	Jacobi et al., 2007	4	Open (2) Forest (2)	*Canga* (4)

*BA, Bahia State; MG, Minas Gerais State; more detailed information can be found in the electronic Supplementary Data.*

**FIGURE 2:**
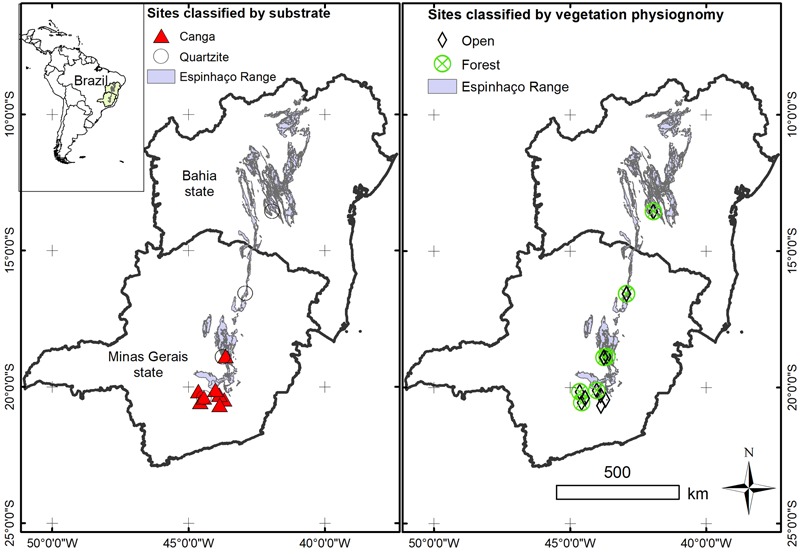
Map showing the geographical location of the sites included in our analysis. Each site is classified according to the substrate and physiognomy of the vegetation.

From the matrix of 47 sites (locations shown in **Figure 2**), we extracted the list of 2920 species (Supplementary Data 1.2, 1.3). We updated the synonymy and formatted our species list using PlantMiner (Carvalho et al., 2010), and the final species list was imported into Phylocom (Webb et al., 2008) using the megatree R20120829 for our analyses. This megatree does not include the families “Turneraceae” and “Peraceae,” thus we transferred species from these families represented on our list to “Passifloraceae” and “Euphorbiaceae,” respectively. In Phylocom we used the bladj script to date the phylogenetic tree we produced for our species list. Hedges and Kumar (2009) and Bell et al. (2010) offered up-to-date estimates of divergence time for Angiosperm on the basis of which Gastauer and Meira-Neto (2016) created an updated “ages” file for phylogenetic analysis and adapted the R20120829 megatree to match the new ages file. We used the enhanced R20120829 megatree and new ages file (based on the estimates of Hedges and Kumar, 2009; Bell et al., 2010) created by Gastauer and Meira-Neto (2016) to build our own “Espinhaço megatree” comprising the 2920 species reported in our collated surveys from the Espinhaço range. During the analysis the parasitic species *Pilostyles blanchetii* was omitted from our megatree due to uncertainty as to the correct phylogenetic placement of this genus, even to the level of order.

### Phylogenetic Reconstruction

The “Espinhaço megatree” showing the evolutionary position of each species relative to the others and the matrix with the presence–absence data, showing the occurrence of each species in each site, were loaded in the R environment to perform multivariate and phylogenetic analyses, performed with Vegan (Oksanen et al., 2017) and Picante (Kembel et al., 2010) packages. Because R does not accept the “-” symbol in the column labels, for species names with composite epithets (*Dolichandra unguis-cati, Clusia burle-marxii, Paepalanthus grao-mogolensis*, etc.), we manually replaced “-” with “.” (e.g., dolichandra_unguis.cati) in our dated megatree (Supplementary Data 3).

### Tree Visualization

We used iTOL (Letunic and Bork, 2016) to visualize the Espinhaço megatree, including highlighting of the major clades included and the occurrence of individual species in different substrates (*canga* or quartzite or both) and in different vegetation physiognomies (open vegetation or forest or both).

### Similarity Analysis

To evaluate the floristic differences between the plant communities in different habitats (FC, FQ, OC, OQ) we calculated a metric of beta diversity and two metrics of phylogenetic alpha diversity using Vegan (Oksanen et al., 2017) and Picante (Kembel et al., 2010) packages. To evaluate beta diversity between sites we excluded species reported only from a single site within our matrix (singletons) and calculated the Bray–Curtis distance among sites. Bray–Curtis (also called Sorensen distance for presence–absence data) is an ecological distance that considers the proportion of shared species among sites as a measure of distance (the larger the number of shared species, the smaller the distance) (Legendre and Legendre, 2012). Using the Vegan function “vegdist()” we created a matrix showing the ecological distance of each site to all other sites. We then used ordination and grouping methods to graphically express the floristic relationships among sites. To group the sites we used the Ward algorithm (as implemented in the ward.D2 argument of “hclust()” in Vegan). To order the sites we used a non-metric multidimensional scaling (NMDS) with two dimensions. NMDS is a multivariate method that finds the “better” relative position of each site with respect to all others based on the available distance matrix (in our case, a Bray–Curtis matrix). We plotted a 95% confidence interval ellipse around each habitat to show their grouping using the “ordiellipse()” function in Vegan.

### Phylogenetic Structure Analysis

We used the 47 site matrix and the Espinhaço megatree to calculate two phylogenetic metrics: the Mean Pairwise Distance (MPD) and the Mean Nearest Taxon Distance (MNTD). To interpret the values of MPD and MNTD we compared the observed values of MPD and MNTD with those obtained using bootstrap analysis of a null model, where we shuffled the tips of the phylogenetic tree 999 times and recalculated values. This generates the Standard Effect Size (SES) for each metric, Net Relatedness Index (NRI) and Nearest Taxon Index (NTI), respectively. At the level of individual sites, values of NRI and NTI larger than 1.96 or smaller than -1.96 are significantly greater than the mean values obtained from the null model and indicate sites with significant phylogenetic clustering or overdispersion, respectively.

Following tests for normality and homogeneity of variance (Supplementary Table 1), to test whether the phylogenetic community structure (as measured by mean NRI and NTI) showed clustering for assemblages in a particular habitat we used one-tailed *t*-tests on the subsets of interest. To evaluate which factors best explain variation in the degree of phylogenetic clustering between communities we used ANOVA, with *post hoc* Tukey tests to determine which means differed significantly from each other (SAS Institute Inc., 2015).

### Identifying Over- and Under-represented Clades

To determine which plant clades were over- or under-represented in particular habitat types and to visualize these results to facilitate interpretation we used nodesig and nodesigl algorithms in Phylocom. Nodesig evaluates for a given tree (showing where each species fits in the evolutionary tree) and a given presence-absence matrix (showing where each species occurs) whether a particular evolutionary lineage is over- or under-represented in each site. Using the iTOL tree viewer we plotted our nodesig outputs for individual sites (Supplementary Data 4.1, 4.2) and for subsets of sites with the same substrate and vegetation physiognomy (FC, FQ, OC, OQ). We tabulated nodes for which at least half the sites of a particular habitat type were over- or under-represented (Supplementary Table 2) and, where possible, attributed names to these clades in order to provide a qualitative description of how the differences in the phylogenetic structure manifest themselves in terms of plants observed at particular sites. For each habitat, estimated crown dates for lineages over-represented on at least half of the sites were tabulated in order to determine which habitats, if any, had a prevalence of relatively old lineages.

## Results

### Overview

**FIGURE 3:**
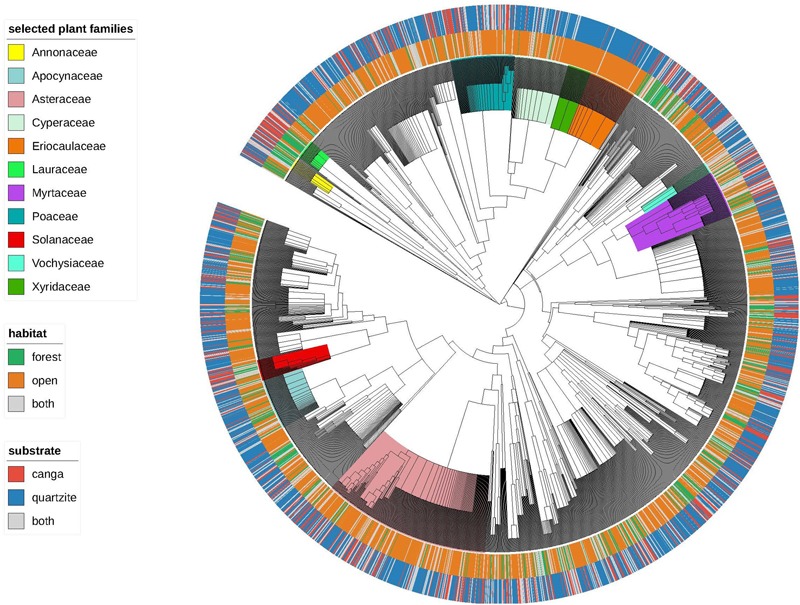
The “Espinhaço megatree” indicating vegetation and substrate affinities for the 2920 species. Plant families mentioned in the results and discussion are highlighted.

Merged species lists for the 47 sites included in our analysis yielded 2920 species representing 789 genera and 135 families, with species recorded per site ranging from 20 to 503 and averaging 148. Our initial visualization (**Figure 3**) suggested strong associations between certain clades and particular substrate-physiognomy combinations and a relatively few and scattered species found on both substrates and/or in both vegetation physiognomies. For example, Xyridaceae and Eriocaulaceae were very strongly associated with OQ while Poaceae, also found mainly in open habitats, had several species reported from both *canga* and quartzite. Groups of species confined to forest habitats on *canga* were infrequent but examples were seen in the early branching angiosperms, e.g., some Annonaceae and Lauraceae. Among the families represented by numerous species, Myrtaceae were notable for having many species reported from both open and forest habitats and/or from both *canga* and quartzite.

### Similarity Analysis

**FIGURE 4:**
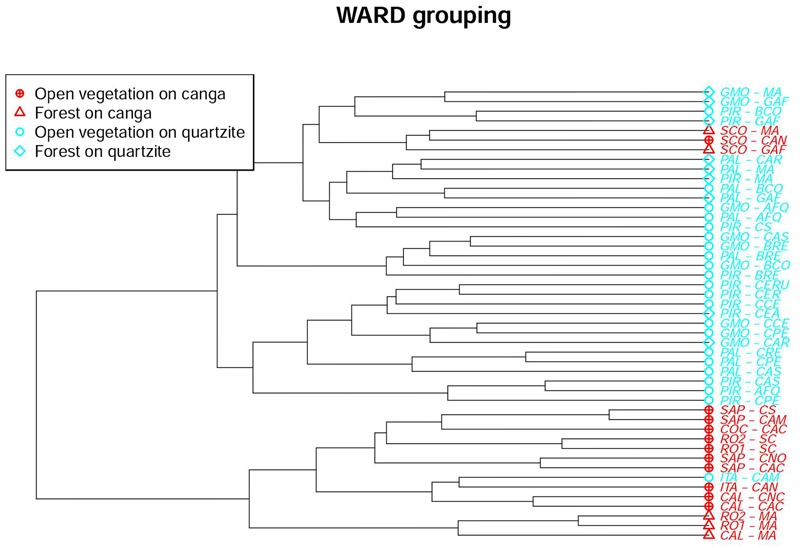
Dendrogram showing results of Ward hierarchical clustering of 47 sites in the Espinhaço range.

**FIGURE 5:**
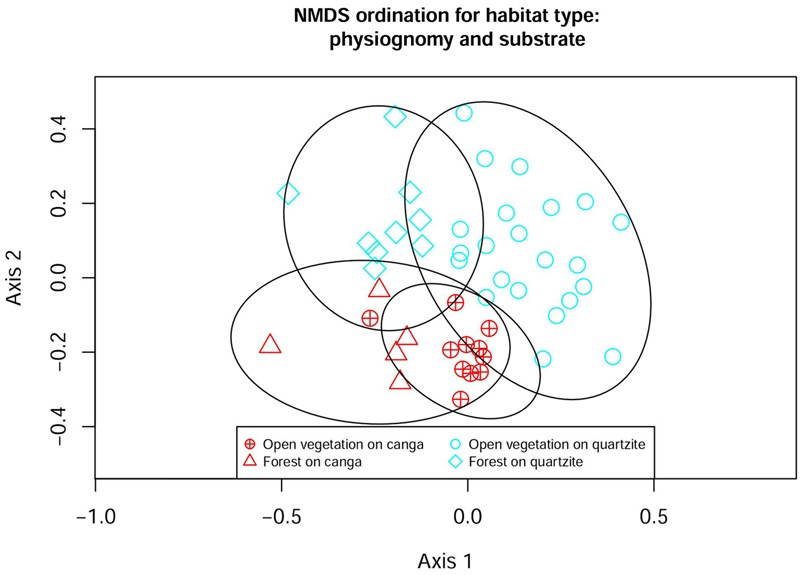
Non-metric multidimensional scaling (NMDS) ordination of 47 sites in the Espinhaco range. Bray–Curtis distance, final stress = 0.23. Ellipses show 95% confidence limits for delimitation of each group.

The Ward similarity analysis illustrated the influence of substrate, physiognomy and location on floristic similarity values. The dendrogram (**Figure 4**) shows a primary division driven by substrate, with all but three of the *canga* sites forming a distinct group that includes just one site on quartzite at ITA. The remaining three *canga* sites (all at SCO) form a cluster which is nested within a major grouping comprising all the quartzitic sites (except the ITA site mentioned above). The next level of grouping appears to be influenced more by physiognomy: within the main *canga* grouping, three forest sites form a sub-group distinct from the remainder, which are from open vegetation. Similarly, the main quartzitic group is divided into two subgroups: one dominated by sites with OQ but including two forest sites on quartzite while the second subgroup is more heterogeneous. The first dichotomy in the heterogeneous subgroup separates a group of quartzite sites with open vegetation from the remaining sites which in turn are divided into four groupings. The composition of the smallest groupings reflects geographical locations, with sites frequently appearing least dissimilar to another site at the same location. The major groupings detected in the Ward similarity analysis were also reflected in the NMDS analysis (**Figure 5**), which classified each site with the corresponding habitat type.

### Phylogenetic Structure Analysis

**FIGURE 6:**
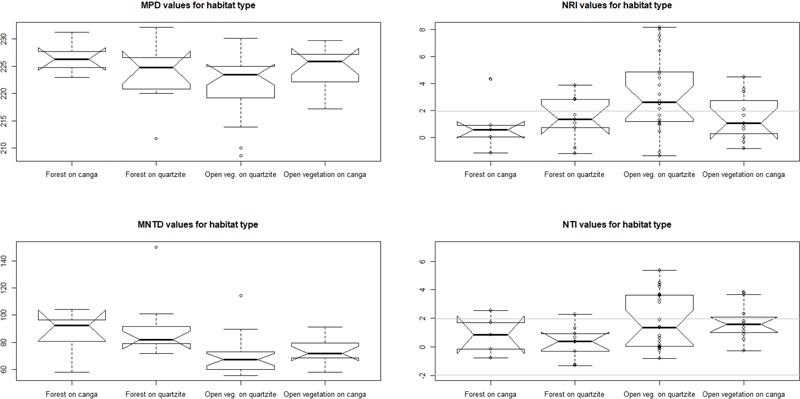
Values of MPD, MNTD and their standardized metrics comparing the observed values against a null model: the NRI and NTI, respectively. Individual sites (dots in the boxplots) with values of NRI and NTI above +1.96 (gray lines) are statistically phylogenetically clustered.

Analysis of phylogenetic structure revealed the general prevalence of phylogenetic clustering in the open *campo rupestre* communities studied (**Figure 6** and **Table 2**). Significant phylogenetic clustering as measured by NRI and NTI was evident in mean values both for open sites on *canga* and for open sites on quartzite. However, for forest sites on *canga*, neither NRI nor NTI showed means significantly greater than zero while forest sites on quartzite showed significant phylogenetic clustering as measured by mean NRI but not by NTI.

**Table 2:** Results of one-tailed *t*-tests ascertaining whether mean NRI and NTI values of assemblages in different habitats (defined by substrate and vegetation physiognomy) differed significantly from zero.

Habitat		Count	mean	*SD*	*SE*	*df*	*t* stat	*p*-value	
Forest on *canga* (FC)	NRI	5	0.92	2.06	0.92	4	0.99696	0.18760	NS
	NTI	5	0.83697	1.35	0.60	4	1.39046	0.11838	NS
Forest on quartzite (FQ)	NRI	9	1.37	1.67	0.56	8	2.45424	0.01984	^∗^
	NTI	9	0.35	1.16	0.39	8	0.89474	0.19852	NS
Open vegetation on *canga* (OC)	NRI	11	1.49	1.73	0.52	10	2.86135	0.00846	^∗∗^
	NTI	11	1.68	1.22	0.37	10	4.54967	0.00053	^∗∗∗^
Open vegetation on quartzite (OQ)	NRI	22	3.28	2.85	0.61	21	5.41118	0.00001	^∗∗∗^
	NTI	22	1.87	1.94	0.41	21	4.51751	0.00009	^∗∗∗^

*^∗^*p* < 0.05, ^∗∗^*p* < 0.01, ^∗∗∗^*p* < 0.001.*

Analysis of variance results suggest that substrate and physiognomy account for some variation in NRI (both marginally significant) while the interaction of substrate and physiognomy was clearly not significant. In contrast, for NTI physiognomy had significant explanatory power (*p* = 0.02) while neither substrate nor the interaction of substrate and physiognomy were significant (**Table 3**). Tukey tests on the results of the ANOVA showed that neither NRI nor NTI values differed significantly between communities on quartzite and those on *canga* (*p* > 0.05 in each case). In contrast, NTI values for communities with open physiognomies were significantly greater than those for forest formations (*p* = 0.0165) while NRI values did not differ significantly between open and forest formations (*p* > 0.05).

**Table 3:** Results of ANOVA to evaluate relative contribution of substrate, physiognomy and interactions of substrate and physiognomy to explaining variation in NRI and NTI.

		*df*	Mean square	*F*-value	*P*r > *F*	
NRI	Substrate	1	21.18	3.81	0.0576	NS
	Physiognomy	1	20.48	3.68	0.0617	NS
	Substrate^∗^physiognomy	1	4.05	0.73	0.3981	NS
	Error	43	5.57			
NTI	Substrate	1	0.01	0.00	0.9847	NS
	Physiognomy	1	16.22	6.23	0.0165	^∗^
	Substrate^∗^physiognomy	1	1.03	0.40	0.5320	NS
	Error	43	2.60			

*^∗^*p* < 0.05.*

### Identifying Over- and Under-represented Clades

**FIGURE 7:**
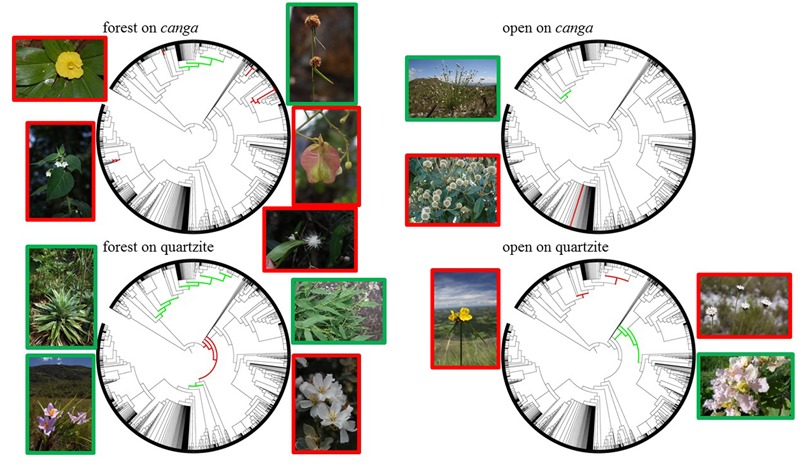
Phylogenetic representations of the “Espinhaço megatree” summarizing over-represented and under-represented clades for each of the four vegetation:substrate types. Clades over-represented in a particular habitat type are shown in red, while under-represented clades are highlighted in green. Images of selected taxa for over-represented (framed in red) and under-represented (framed in green) clades are placed adjacent to each phylogeny as follows: forest on *canga* – *Chamaecostus subsessilis* (top left, photo by B. Klitgaard), *Solanum didymum* (bottom left, photo by W. Milliken), *Rhynchospora exaltata* (top right, photo by W. Milliken), *Serjania paradoxa* (middle right, photo by D. Zappi), *Myrcia venulosa* (bottom right, photo by W. Milliken); open on *canga* – *Paepalanthus erectifolius* (top left, photo by D. Zappi), *Eremanthus incanus* (bottom left, photo by P. L. Viana); forest on quartzite – *Paepalanthus planifolius* (top left, photo by W. Milliken), *Vellozia glabra* (bottom left, photo by W. Milliken), *Panicum sellowii* (top right, photo by W. Milliken), *Trembleya laniflora* (bottom right, photo by D. Zappi); open on quartzite – *Cephalostemon riedelianum* (left, photo by W. Milliken), *Paepalanthus comans* (top right, photo by D. Zappi), *Banisteriopsis malifolia* (bottom right, photo by W. Milliken).

Clades indicated by nodesig as being significantly over- or under-represented in at least half of the sites from a particular habitat type are shown in **Figure 7**. The families Solanaceae and Myrtaceae (especially the genus *Myrcia*) were over-represented in forest sites on *canga* while the cyperid and xyrid monocots and consecutive monocot clades above them tended to be under-represented in these sites. In contrast, FQ bore assemblages in which Eudicots were over-represented, specifically Pentapetalids, while Narthecidae and several other monocot clades were under-represented. Open vegetation on iron showed an over-representation of the genus *Eremanthus* (Asteraceae) and under-representation of the xyrid monocots. Commelinids and cyperids + xyrids were over-represented in OQ while Rosids and Subrosids were under-represented. Tabulation of the estimated crown ages for these clades (**Table 4**) showed that clades which are significantly over-represented in assemblages on *canga* generally represent younger lineages than those which are significantly over-represented in assemblages on quartzite. The oldest over-represented clades (>100 Mya) are all over-represented in FQ while the three youngest are all over-represented on FC sites (≤37 Mya).

**Table 4:** Clades which are significantly over-represented in at least half of the sites of a particular habitat and their estimated ages.

Habitat type	Over-represented Family/genus	Over-represented clade of other rank	Estimated age (Millions of years)
*Forest on canga*	*Myrcia* (Myrtaceae)		26.1
*Forest on canga*		Solanaceae *pro parte* incl. *Solanum, Nicotiana, Brunfelsia, Cestrum, Schwenckia*	<37
*Forest on canga*	Solanaceae		37
*Open vegetation on canga*	*Eremanthus* (Asteraceae)		20
*Forest on canga*	Myrtaceae		52.2
*Forest on canga*		Myrtaceae + Vochysiaceae	59.7
*Forest on canga*		‘meliaceae_to_rutaceae’ + Sapindaceae	66.4
*Open vegetation on quartzite*		(cyperaceae_to_juncaceae + mayaca) + (eriocaulaceae + xyridaceae)	67
*Forest on canga*		commelinales+ (*costus*+*hedychium*)	83
*Open vegetation on quartzite*		Commelinids	96
*Forest on quartzite*		Pentapetalids	116.9
*Forest on quartzite*		Sabiales _to _Asterales	126
*Forest on quartzite*		Eudicots	128.9

*Crown ages are extracted from the exponential model (Gastauer and Meira-Neto, 2016) used to build the Espinhaco megatree. More detailed information can be found in Supplementary Table 2.*

## Discussion

Analysis of environmental impacts on phylogenetic structure have largely focused on broad-scale effects, such as climate. The present study is one of the few that have considered the role of soil substrate and vegetation physiognomy, two factors of critical importance to plant distributions. The use of environmental contrasts (geologically different substrates; open versus forested vegetation) provides a useful framework for investigation of the impact of environment on patterns of biodiversity. Moreover, our study is the most comprehensive study of community phylogenetic structure in *campo rupestre* to date and the only one to include sites on *canga*, a substrate that is a target for extractive industry and thus one of the most threatened habitats in Brazil (Jacobi and Carmo, 2008). Our aim was to explore variation in floristic and phylogenetic diversity and structure across different *campo rupestre* assemblages on contrasting rock formations within the Espinhaço range of Eastern Brazil and to explore whether the relative ages of lineages which are significantly over-represented in these assemblages provide support for the Gondwanan Heritage Hypothesis of Ocbil theory. We found evidence of distinct floristic assemblages in each of the four habitat types studied and detected significant phylogenetic structure in three of the four habitats, the exception being forest on *canga* (FC). FC sites were also notable for an over-representation of clades representing relatively young lineages consistent with expectations for young, often disturbed, fertile landscapes (Yodfels sensu Hopper et al., 2016) compared to those on quartzite which had an over-representation of clades from relatively old lineages as predicted for an Ocbil.

Similarity analysis clearly showed the importance of substrate to floristic composition, with most communities on *canga* having a flora more similar to that of other sites on *canga* than to the majority of sites on quartzite. The NMDS ordination also showed the importance of substrate, but physiognomy appeared even more important, with only one site crossing an otherwise clear separation between the floras of forest and open vegetation. Considered in combination, these results based on floristic composition provide strong support for our *a priori* recognition of four habitat types based on substrate and physiognomy: FC, FQ, OC and OQ. This is of great importance from a biodiversity perspective. The Espinhaço has substantial environmental heterogeneity related to both substrate, altitude, topographic position and microrelief. While the windward face of a mountain can harbor forests, the top of the same mountain bears *campo rupestre*, with forests restricted to small depressions where deeper soils accumulate. At a landscape scale, we can see sharp transitions between vegetation types and, as we see in our analysis, each habitat type has its own flora with its own environmental processes shaping its composition, in terms not only of individual species, but also of lineages.

We found phylogenetic clustering to be common in *campo rupestre* communities on both *canga* and quartzite and in both open and forest physiognomies, but the degree of phylogenetic clustering detected differed between the habitat types and with the metric used. Phylogenetic clustering was most evident in open assemblages, with both OC and OQ having mean NRI and NTI significantly different from zero, indicating significant phylogenetic clustering both near the tips of the phylogeny (NTI) and also involving the deeper nodes in the tree (NRI). Phylogenetic clustering was particularly pronounced in open assemblages on quartzite (OQ) with mean NRI and NTI values exceeding those reported by Miazaki et al. (2015) for angiosperms in open *campo rupestre* vegetation on quartzite at two sites in Minas Gerais and by Pugliesi and Rapini (2015) for Apocynaceae assemblages in the Chapada Diamantina, in the northern and drier part of the Espinhaço, largely covered by open *campo rupestre* formations associated with quartzitic outcrops. [In forest communities, however, NTI was not significantly different from zero on either substrate while mean NRI differed significantly from zero in FQ but not in FC].

Following Cavender-Bares et al. (2009), our results could be interpreted as likely due to the effects of environmental filtering, in an ecosystem where niche conservatism is prevalent. The congruence of our results for OQ with those of earlier authors could be argued to support the impact of relatively strong environmental filters inferred by Miazaki et al. (2015) and Pugliesi and Rapini (2015) and earlier documented by Negreiros et al. (2014) for non-woody species in open *campos rupestres* where a clear pre-dominance of stress-tolerant strategies was reported [and the few species with a greater level of competitiveness had relatively low abundance]. However, recent reviews of the application of phylostructure metrics to community ecology have cautioned against the use of phylogenetic patterns as proxies for community assembly mechanisms (Gerhold et al., 2015) and warned that the metaphor of the environmental filter has likely resulted in overstating the role of abiotic tolerance in shaping community structure (Kraft et al., 2015). These latter authors argue for a stricter application of the term environmental filtering, restricting it to situations where the abiotic environment can be demonstrated to prevent establishment or persistence in the absence of biotic interactions. Since experimental data of this kind are lacking for the vast majority of species in our Espinhaco megatree analysis, we cannot aspire to apply this stricter definition in the discussion that follows. Instead we exercise caution in discussing our results, indicating where evidence suggests there may be a role for environmental filtering but accepting that other potential equally valid explanations have not been explored.

Overall, phylogenetic structure in *campo rupestre* is influenced more by physiognomy than by substrate. Unpacking the relative importance of substrate and physiognomy and their interaction on our two measures of phylogenetic structure, analysis of variance showed that vegetation physiognomy was highly significant in explaining variation in NTI, while substrate and physiognomy were of equal importance (each only marginally significant) in explaining variation in NRI. Surprisingly, substrate x physiognomy interactions did not explain significant variation in either metric. The significance of physiognomy in explaining variation in NTI in our study is consistent with the observations that across *campo rupestre* the development of forest tends to occur in areas that are more favorable in terms of water and/or nutrient availability (Ferrari et al., 2016) and that in these fragmented *campo rupestre* landscapes the co-existence of forests and grasslands at the same altitude indicates that the distribution of tree species might be attributable to additional factors such as soil physical and chemical parameters (Coelho et al., 2018). In this context the lack of phylogenetic clustering of terminals in phylotrees representing assemblages from forest environments may be attributable to a relatively smaller role for environmental filtering in forests than in more environmentally stressful areas where open vegetation develops. Within a series of forest islands embedded in open *campos rupestres* on quartzite, Coelho et al. (2018) showed that high soil fertility and canopy cover were associated with greater height and basal area of trees, suggesting a relaxation of abiotic filters in the less harsh environment which supported forest, which could be consistent with the lack of signal in NTI observed in our forest samples.

Phylogenetic clustering was most evident in OQ, and the clade-by-clade results generated by nodesig revealed over-representation in at least half of the OQ sites in our study of two major clades of monocots: the larger being the commelinid monocot clade, which includes the Poales and three other orders of monocots, a result consistent with the wide diversity of different types of open vegetation encompassed within our OQ study sites. The smaller over-represented clade in OQ is nested within Poales, and comprises the cyperid clade (Cyperaceae, Juncaceae and *Mayaca*) together with the xyrid clade (Eriocaulaceae + Xyridaceae). These latter families include ‘everlasting’ flowers from the iconic megadiverse genera *Paepalanthus* and *Xyris*, long considered emblematic of open *campo rupestre* communities and an important source of income for rural, low-income families (Giulietti et al., 1996). The over-representation of these two monocot clades in OQ may be primarily attributable to niche conservatism with respect to life form, since monocots are mostly herbaceous species more prevalent in open habitats. However, our analysis shows that the xyrid clade is significantly under-represented in most study sites for OC, suggesting a difference between OQ and OC habitats attributable to factors other than life-form. One possible explanation lies in the nutrient-acquisition strategies of Eriocaulaceae and Xyridaceae. In their pioneering study of mineral nutrition strategies of *campo rupestre* plants, Oliveira et al. (2015) studied 50 species on quartzite- and arenite-derived substrates, and reported a range of root specializations associated with *campo rupestre* soils which are comparable to the most phosphorus-impoverished soils in the world. Roots covered in very fine root hairs over their entire length were reported for 32 species, most of them in the Eriocaulaceae and Xyridaceae, but also in Asteraceae. The authors invoke convergent evolution and Ocbil theory in interpreting the striking similarities in patterns of nutrient-acquisition strategies between *campos rupestres*, kwongan and fynbos, concluding that *campos rupestres* are home to several very old Gondwanan lineages which have evolved (*in situ*) a wide range of root specializations to survive P limitation. The ages of the two monocot clades which are over-represented in OQ sites are 67 and 96 Mya, respectively (**Table 4**), lending further support to the interpretation of open *campo rupestre* systems on quartzite as Ocbils. In contrast to the monocot over-representation in OQ sites, the only clade that our study showed to be over-represented in most OC sites is the genus *Eremanthus* (estimated age c. 20 Mya). These tree-like, woody Asteraceae are characteristic of the transition between forest and open habitats on canga, often forming homogenous stands known as ‘*candeias*.’

The cyperid + xyrid clade, so characteristic of OQ, is significantly under-represented in most of the study sites for FC and for FQ. FQ, was revealed by nodesig to have significant over-representation of the early branching clades in our Espinhaço megatree: specifically the Eudicot clade, the clade comprising Sabiales to Asterales and the pentapetalids. These three clades represent the oldest lineages which are significantly over-represented in any habitat in our study (**Table 4**), a result consistent with the inclusion of forest assemblages on quartzite within the *campo rupestre* Ocbil. While potentially attributable to niche conservatism of lifeform, this result is also consistent with our tree-wide phylostructure metrics for which phyloclustering was detected by mean NRI (reflecting relatively deep nodes) but not at the level of NTI and may reflect the relative isolation of most FQ sites from the humid forest biome. In a study of forest islands on quartzite in the Serra do Cipó, Coelho et al. (2018) described dispersal of tree species to mountain top forest patches via gallery forests on their eastern slopes but our FQ sites were, for the most part, embedded in the savannas of the Cerrado biome and or, in the case of the more northern sites, in the semi-arid Caatinga biome.

Our tree-wide metrics detected no significant phylogenetic structure across study sites for FC, with neither mean NRI nor mean NTI differing significantly from zero, but clade-by-clade analysis indicated significant over-representation of several dicot clades in FC. These included: the family Solanaceae and a subclade within it encompassing *Solanum* and closely related genera; a suborder of Sapindales soon to be recognized as ‘core Sapindales’ (J. R. Pirani personal communication); the clade formed by Myrtaceae together with Vochysiaceae, and nested within, the family Myrtaceae and the genus *Myrcia.* The prominence of *Solanum* and Solanaceae in FC is consistent with qualitative and quantitative descriptions of *canga* vegetation by earlier authors (Jacobi et al., 2007; Mourão and Stehmann, 2007). The importance of Myrtaceae and, specifically *Myrcia*, has also been highlighted previously, though not to the same extent, and may reflect the geographic location of the FC sites which are all close to the megadiverse Atlantic Forest biome (Stehmann et al., 2009; Brazil Flora Group [BFG], 2015) in which *Myrcia* is exceptionally diverse (Murray-Smith et al., 2009; Lucas et al., 2011; Nic Lughadha et al., 2012; Lucas and Bünger, 2015). Further evidence that proximity to the megadiverse Atlantic Forest biome may be a source of species from genera not commonly associated with *campos rupestres* is provided by the fact that three of the five genera which are most diverse in the Atlantic Forest in Brazil (*Eugenia, Croton* and *Solanum*) are represented by multiple species in FC, but not elsewhere in our study. Notably, the six clades reported above as significantly over-represented in the majority of FC sites are among the seven youngest over-represented lineages in our study (see **Table 4**).

Two very recent, synthetic studies of *campo rupestre* soils (Ferrari et al., 2016; Schaefer et al., 2016) have been of particular interest in the interpretation of our results because they include detailed comparisons of *campo rupestre* on quartzite and on *canga*. Using a conceptual framework very similar to the present study, Ferrari et al. (2016) monitored thermic and hydric dynamics of soils in four sites, contrasting open *campo rupestre* vegetation on *canga* (OC) and FC with open *campo rupestre* vegetation on quartzite (OQ) and FQ. Their results confirm the observations of many earlier authors (Meguro et al., 1996a,b), that soil depth is key, in both quartzite and *canga* landscapes, with forest occurring on much deeper soils than grassland. Furthermore, detailed monitoring of diurnal and seasonal changes in soil moisture showed that, in general, there was greater seasonal variation in soil moisture on *canga*, especially at the surface. *Campo rupestre* grasslands on *canga* (OC) and on quartzite (OQ) showed greater variation in soil moisture than did forest sites, with recorded moisture levels frequently indicating severe water deficits in grassland on *canga* (OC) and even more frequently in grassland on quartzite (OQ), a difference which Ferrari et al. (2016) attribute to the greater clay and silt content of the *canga* soils resulting in greater water retention and availability. No water deficits were recorded for FQ while interpretation of results for FC was complicated by technical/methodological issues. These results suggest that soil moisture factors may represent particularly strong filters in OQ.

Parallel monitoring of air and soil temperatures showed different patterns across the four sites compared by Ferrari et al. (2016). In general, air temperatures of grasslands were similar to those of forest, but soil temperatures in forest more closely reflected air temperature while grassland soil temperatures showed more seasonal and annual variation. The range of this variation is much greater in grassland on *canga* (OC) and soil temperature usually exceeds air temperature to a greater extent than in grassland on quartzite (OQ), a difference which the authors attribute to substrate albedo: dark, red, dense *canga* absorbing more energy than light, pale quartzite. Furthermore, the frequency and maximum duration of events in which soil temperature reached >35°C was highest in grassland on *canga* (OC). Surprisingly, events where the soil temperature exceeded 35°C were also recorded in FQ, albeit with lower frequency and duration than in grassland on *canga* (OC). Thus soil temperature factors may represent particularly strong filters in OC but also, secondarily in FQ.

A cluster analysis of soil profiles from high altitude rocky complexes across Brazil (Schaefer et al., 2016), distinguished two main types of *campo rupestre* soils differing more in physical than in chemical attributes: sandy soils with greater levels of exchangeable Al^3+^, associated with granite/gneiss and quartzite substrates as compared to soils with greater accumulation of organic matter and clayey/silty textures found on *canga* and itabirite. These authors conclude that the low biomass of *campo rupestre* vegetation in general is attributable to low nutrient levels, especially to low phosphorus (P) rather than to metal toxicity. They report P amounts to be particularly critical for vegetation on *canga* from mining areas including those in the Iron Quadrangle of Minas Gerais though, puzzlingly, the lowest P levels they report for any site within our study area are actually for a quartzite site within the Quadrilátero Ferrífero.

In summary, low nutrient levels are prevalent across *campo rupestre*, particularly acute in quartzitic soils, relative to those on *canga*, and a more significant constraint to growth than metal toxicity. Other factors such as soil moisture, water deficits and very high temperatures vary in frequency and intensity across the four habitat types studied and are variously reported to be more severe in grassland (on OQ and/or OC in the case of water deficits and soil temperatures) or FQ (soil temperatures). In these studies no single potential environmental filter was reported to be most severe for FC, a result congruent with the lack of phylogenetic clustering detected in this habitat. Together these considerations may suggest that this assemblage is less likely to be a result of relatively severe environmental filtering than others in our study, though of course in practice environmental factors need to be considered in combination rather than individually in order for their filtering effects to be understood.

Considered as a whole and in the context of the several recent studies on *campo rupestre* flora and soils on quartzite and *canga* substrates, our results on phylogenetic structure, ages of over-represented clades in each habitat and the identities of these clades, lend support to the recognition of both open and forest assemblages on quartzite as Ocbils, with their significant over-representation of clades from several ancient lineages consistent with Gondwanan Heritage Hypothesis predictions (Hopper et al., 2016; Silveira et al., 2016). In contrast, all the evidence suggests that forest assemblages on *canga* represent Yodfels.

Analysis of phylogenetic structure of communities has proved a valuable tool in exploring contemporary ecological interactions and in linking community ecology with biogeography and evolution (Vamosi et al., 2009) and with conservation and macroecology (Tucker et al., 2017). Any attempt at synthesis on the scale of the study reported here presents significant challenges in terms of data standardization and quality control, choice of appropriate analytical approaches and interpretation of results. Vamosi et al. (2009) provide a useful checklist for evaluating phylogenetic community structure analyses. We consider the broad taxonomic scope (all angiosperms), broad geographical coverage, attention to taxonomic consistency and data integrity to be particular strengths of our study. However, we acknowledge and explain limitations in certain other aspects which may be addressed in future studies.

Although beyond the scope of the present study, a useful next step would be to collate information on regional species pools, an approach that has proven effective in understanding the historical relationships among ecologically similar sites across a continental scale (e.g., DRYFLOR et al., 2016; Dexter et al., 2017). Our species pool (metacommunity) was the combined taxon list for all the sites included in our study. An alternative approach would be to use a published list of species reported from *campo rupestre* s.l. (Brazil Flora Group [BFG], 2015). This would result in a species pool >60% larger than that we analyzed, or up to five times larger if we allowed for the possibility of Atlantic Forest species forming part of *campo rupestre* assemblages. However, this larger species pool would present greater challenges for data management and analysis and would also greatly reduce the proportion of the species pool represented in any single site, with potential detrimental effects on the statistical power to detect phylogenetic structure. Kraft et al. (2007) concluded on the basis of simulation studies that local communities comprising 30–60% of the regional pool would offer greatest statistical power. Vamosi et al. (2009) showed that, in practice, few published studies fall within this range, with most having mean local richness less than 30% of the total regional species pool, and our study is no exception.

Our study provides insights for those concerned with the conservation and sustainable management of areas of *campo rupestre*, including those on *canga* which are currently the subject of exceptional pressure from mining interests. The clear floristic distinctions between the four habitat types considered are a pointer to the importance of adequate representation of each type (and sub-types thereof where they are recognized) in protected area networks and sustainable management plans. Our conclusion that the forest communities on *canga* are quite different in phylogenetic structure from other *campo rupestre* assemblages and should in fact be interpreted as Yodfels requires further exploration before it can be considered sufficiently robust to inform appropriate conservation measures, which differ greatly between Ocbils and Yodfels (Hopper et al., 2016). However, the possibility that these forests on *canga* may harbor diverse subsets of Atlantic Forest diversity could lead to the identification of win–win approaches, whereby the vegetation restoration projects which are a legal requirement following mine closure (Skirycz et al., 2014) could also represent opportunities to safeguard and even increase populations of some of the most threatened species of the Atlantic Forest.

To our knowledge, this study is one of the most inclusive to date to focus primarily on non-forest ecosystems. However, increasing adoption of a very broad definition of ‘Rupestrian Grasslands’ to include all high altitude rocky complexes in Brazil highlights the opportunity for an even more inclusive analysis including other rupestrian environments associated with highlands, such as the tepuis of Roraima and the *cangas* of Carajás in the Brazilian Amazon. Increased investment in floristic inventory in these areas over the past decade is resulting in the publication of site-based lists suitable for inclusion in such studies (Viana and Lombardi, 2007; Nadruz-Coelho et al., 2015; Barbosa-Silva et al., 2016).

As more comprehensive data on species distributions becomes better understood and accessible, covering other rupestrian environments on highlands in different parts of the continent, studies such as the present one will elucidate processes underlying biodiversity dynamics at ever-increasing spatial scales and ecological heterogeneity.

## Author Contributions

DZ co-designed and initiated this study, collated the plot data used in the analysis, contributed to integration and interpretation of the data, and contributed to the final manuscript. MM contributed to collation of the plot data used in the analysis, conducted and documented the bulk of the similarity and phylogenetic analyses, contributed to integration and interpretation of the results, and contributed to the final manuscript. TM conducted statistical analysis, contributed to integration, visualization and interpretation of the results, also contributing toward the final manuscript. EN co-designed this study, contributed to statistical analysis and to integration, and interpretation of the results, and played a lead role in drafting the final manuscript. All authors have approved the final version to be published and agree to be accountable for all aspects of the work, ensuring questions related to its accuracy and integrity are appropriately investigated and resolved.

## Conflict of Interest Statement

The authors declare that the research was conducted in the absence of any commercial or financial relationships that could be construed as a potential conflict of interest.
