# Supplementary material for: Plant Biodiversity Drivers in Brazilian Campos Rupestres: Insights from Phylogenetic Structure
Source: Front Plant Sci. 2017 Dec 19;8:2141. doi: 10.3389/fpls.2017.02141 (PMC5742226; doi:10.3389/fpls.2017.02141)
Supplement: Supplementary file 1 [file Table_1.pdf]

Supplementary Material

Plant biodiversity drivers in Brazilian *campos rupestres*: insights from phylogenetic structure.

Daniela C. Zappi*, Marcelo Freire Moro, Thomas R. Meagher, Eimear Nic Lughadha

*** Correspondence:** Daniela C. Zappi: daniela.zappi@itv.org

1 Supplementary Data

The following Supplementary Data files are available through the Figshare repository under https://doi.org/10.6084/m9.figshare.5480536.v1

Supplementary Data 1.1- Matrix species-sites initial dataset with 66 sites

Supplementary Data 1.2- Matrix species-sites with selected 47 sites

Supplementary Data 1.3- taxa

Supplementary Data 2.1- agesexp by Gastauer & Meira-Neto 2016

Supplementary Data 2.2- megatree R20120839mod – Gastauer & Meira-Neto 2016.new

Supplementary Data 3- espinhaco.tree-dated

Supplementary Data 4.1- nodesigl-results

Supplementary Data 4.2- nodesigresults

Supplementary Data 5- table.phylodist-47sites

2 Supplementary Figures and Tables

Supplementary Table 1. Tests for normality and homogeneity of variance for NRI and NTI.

	Shapiro Wilk test for normality		Levene's test for homogeneity of variance		
	W	p-value	Df	F value	Pr(> F)
NRI	0.97026	0.2711	3, 43	1.6264	0.1973
NTI	0.95913	0.09941	3, 43	3.0233	0.03979

Supplementary Table 2 Nodes in the phylogeny with significantly more/less daughter taxa in at least half of the sites of that habitat type compared with a random draw of taxa from the supertree (Phylocom procedure nodesig; Webb et al. 2008). [Over-represented clades included in Table 4 in the main paper are shaded. Clades without accepted names are listed in parentheses.] Where the significant node occurs below the family level, the family name follows it, for reference. Where the significant node occurs above the family level the clade name appears. Sig. more/less: significantly more or less daughter taxa in the given age category.

**Habitat type**	**Sig. more/ less**	**Node id**	**Family/ genus**	**Clade**	**Crown ages extracted from the exponential model (Gastauer & Meira-Neto 2016) used to build the Espinhaco megatree (Mya)**
Forest on canga	More	346	Solanaceae		37
	More	347		Solanaceae *pro parte* incl. *Solanum, Nicotiana, Brunfelsia, Cestrum, Schwenckia*	< 37
	More	1440		'meliaceae_to_rutaceae' + Sapindaceae	66.4
	More	1814		Myrtaceae + Vochysiaceae	59.7
	More	1815	Myrtaceae		52.2
	More	1896	*Myrcia* (Myrtaceae)		26.1
	More	3433		commelinales+ (*costus*+*hedychium*)	83

Forest on canga	Less	3011		(((cyperaceae_to_juncaceae + *mayaca*) + (eriocaulaceae +xyridaceae)) + poaceae) + *cephalostemon*	79
	Less	3012		((cyperaceae_to_juncaceae + mayaca) + (eriocaulaceae + xyridaceae))+poaceae	73
	Less	3013		(cyperaceae_to_juncaceae + mayaca) + (eriocaulaceae + xyridaceae) i.e. cyperid clade + xyrid clade	67
Forest on quartzite	More	3		Eudicots	128,9
	More	4		Sabiales _to _Asterales	126
	More	5		Pentapetalids	116,9
Forest on quartzite	Less	2715		Narthecidae	118
	Less	2736		narthecidae minus alismatales	112.5
	Less	2737		Missing clade name	
	Less	2750		?Asparagales + Commelinids?	101.5
	Less	3011		(((cyperaceae_to_juncaceae + mayaca) + (eriocaulaceae + xyridaceae))+poaceae)+cephalostemon	79
	Less	3012		((cyperaceae_to_juncaceae + mayaca) + (eriocaulaceae + xyridaceae))+poaceae	73
	Less	3013		(cyperaceae_to_juncaceae + mayaca) +	67

				(eriocaulaceae + xyridaceae)	
	Less	3133		eriocaulaceae + xyridaceae = xyrid clade	50.25
Open vegetation on canga	More	985	*Eremanthus* (Asteraceae)		20
Open vegetation on canga	Less	3133		eriocaulaceae + xyridaceae = xyrid clade	50.25
Open vegetation on quartzite	More	2931		Commelinids	96
	More	3013		(cyperaceae_to_juncaceae + mayaca) + (eriocaulaceae + xyridaceae)	67
Open vegetation on quartzite	Less	1415		Subrosids	116.8
	Less	1416		Rosids	108
	Less	1417		?Eurosids?	
